# Radiogenomic Features of GIMAP Family Genes in Clear Cell Renal Cell Carcinoma: An Observational Study on CT Images

**DOI:** 10.3390/genes14101832

**Published:** 2023-09-22

**Authors:** Federico Greco, Andrea Panunzio, Alessandro Tafuri, Caterina Bernetti, Vincenzo Pagliarulo, Bruno Beomonte Zobel, Arnaldo Scardapane, Carlo Augusto Mallio

**Affiliations:** 1 Department of Radiology, Cittadella Della Salute, Azienda Sanitaria Locale di Lecce, Piazza Filippo Bottazzi, 2, 73100 Lecce, Italy; 2 Department of Urology, “Vito Fazzi” Hospital, Piazza Filippo Muratore, 1, 73100 Lecce, Italy; panunzioandrea@virgilio.it (A.P.); tafuri.alessandro@gmail.com (A.T.); enzopagliarulo@yahoo.com (V.P.); 3 Department of Medicine and Surgery, Fondazione Policlinico Universitario Campus Bio-Medico, Via Alvaro del Portillo, 200, 00128 Roma, Italy; c.bernetti@unicampus.it (C.B.); b.zobel@policlinicocampus.it (B.B.Z.); c.mallio@policlinicocampus.it (C.A.M.); 4 Research Unit of Radiology, Department of Medicine and Surgery, Università Campus Bio-Medico di Roma, Via Alvaro del Portillo, 21, 00128 Roma, Italy; 5 Dipartimento Interdisciplinare di Medicina, Sezione di Diagnostica Per Immagini, Università degli Studi di Bari “Aldo Moro”, Piazza Giulio Cesare, 11, 70124 Bari, Italy; arnaldo.scardapane@gmail.com

**Keywords:** clear cell renal cell carcinoma, computed tomography, genes, GIMAP, imaging, immunocytes, immunotherapy, precision medicine, radiogenomics, radiologic features

## Abstract

GTPases of immunity-associated proteins (GIMAP) genes include seven functional genes and a pseudogene. Most of the GIMAPs have a role in the maintenance and development of lymphocytes. GIMAPs could inhibit the development of tumors by increasing the amount and antitumor activity of infiltrating immunocytes. Knowledge of key factors that affect the tumor immune microenvironment for predicting the efficacy of immunotherapy and establishing new targets in ccRCC is of great importance. A computed tomography (CT)-based radiogenomic approach was used to detect the imaging phenotypic features of GIMAP family gene expression in ccRCC. In this retrospective study we enrolled 193 ccRCC patients divided into two groups: ccRCC patients with GIMAP expression (*n* = 52) and ccRCC patients without GIMAP expression (*n* = 141). Several imaging features were evaluated on preoperative CT scan. A statistically significant correlation was found with absence of endophytic growth pattern (*p* = 0.049), tumor infiltration (*p* = 0.005), advanced age (*p* = 0.018), and high Fuhrman grade (*p* = 0.024). This study demonstrates CT imaging features of GIMAP expression in ccRCC. These results could allow the collection of data on GIMAP expression through a CT-approach and could be used for the development of a targeted therapy.

## 1. Introduction

In recent years many fields of biomedical research have evolved due to the availability of genomic information provided by the open-source data of the Human Genome Project [1,2]. Renal cell carcinoma genome sequencing has detected numerous mutations of prognostic value. Due to these advances in genomics, a considerable interest in correlating these data with imaging characteristics has grown [3,4,5]. This interest has resulted in a new field of research called radiogenomics. This field combines imaging phenotypes, macroscopic manifestations detected by imaging processes occurring at the molecular level, and genomic of diseases (i.e., gene expression patterns, gene mutations, and other genome-related features) [6,7]. Radiogenomics obtains data on the whole tumor, in contrast to genomic tests carried out on biopsy specimens [8]. This is one of the greatest advantages of radiogenomics as data collection on biopsy specimen analyzes both gene expression and gene mutations on small samples only and not on the entire tumor lesion, possibly missing data on the entire heterogeneity of the disease, which is typical in clear cell renal cell carcinoma (ccRCC) [8]. Furthermore, radiogenomics can overcome the obstacle that tumor cells with a similar genotype can show different phenotypes allowing the evaluation of the relationship between genomic data and body composition assessed by means of computed tomography (CT)-approach in ccRCC patients [9,10,11].

The tumor immune microenvironment (TIME) is correlated with effect of immunotherapy and clinical outcomes in malignancies [12,13,14]. Knowledge of key factors that affect the tumor immune microenvironment for predicting the efficacy of immunotherapy and establishing new targets in ccRCC is of great importance.

Human GTPases of immunity-associated proteins (GIMAP) family genes span approximately 500 KB on chromosome 7 and include seven functional genes (GIMAP1, GIMAP2, GIMAP4, GIMAP5, GIMAP6, GIMAP7, GIMAP8) and a pseudogene [15]. The GIMAPs are similar in the N-end sequence and possess a guanine nucleotide-binding domain called GTPase [15,16]. Most of the GIMAPs participate in the maintenance and development of lymphocytes. The activities of GIMAP proteins are summarized in Table 1 [17,18,19,20,21]. GIMAPs could inhibit the development of tumor by increasing the amount and antitumor activity of infiltrating immunocytes. To date, CT signs related to GIMAP expression in ccRCC patients have not been evaluated. The aim of this study is to investigate the imaging phenotype of GIMAP expression in ccRCC patients. Specifically, we hypothesized that ccRCC with GIMAP expression may show specific CT radiogenomic features.

## 2. Materials and Methods

### 2.1. The Cancer Genome Atlas

The Cancer Genome Atlas (TCGA), funded by the National Cancer Institute and the National Human Genome Research Institute (NHGRI), is an atlas of genetic changes in more than 20 types of cancer, including ccRCC. Tissue samples, submitted from all participating institutions, after obtaining institutional review board approval, were subjected to complete multiplatform genomic characterization and analysis. The Cancer Imaging Archive, a National Cancer Institute-supported anonymized image repository, was used to upload, in DICOM format, the pretreatment imaging data. Medical images and tissue samples from the TCGA are linked by a unique identifier and are accessible for public download [22].

### 2.2. ccRCC Patients

A total of 267 patients with histological diagnosis of ccRCC were retrospectively analyzed between November 2019 and February 2020 and enrolled based on medical history, CT images, and exclusion criteria. The cohort was selected from consecutive patients with ccRCC undergoing CT for disease staging.

Exclusion criteria were: congenital solitary kidney, previous renal ablation, and heminephrectomized and nephrectomized patients; these patients were excluded because of previous interventions, procedures, or congenital anatomical variants. Moreover, cirrhotic patients, patients who had undergone chest CT only or magnetic resonance imaging examination only, and patients with incomplete imaging dataset were also excluded.

### 2.3. Imaging Features

The following basic radiologic features were analyzed for each tumor: size (in mm), composition (solid or cystic), margin (well-defined or ill-defined), necrosis (detected only for solid tumors: 0%, 1–33%, 34–66% or >66%), calcification (absent or present), and growth pattern (endophytic, <50% exophytic, or ≥50% exophytic) [23]. Endophytic growth pattern was considered when the whole tumor was localized within the renal parenchyma, exophytic growth pattern was defined as less than 50% of the tumor localized outside the renal parenchyma, while exophytic growth pattern ≥50% was considered when at least 50% of the tumor was located outside the renal parenchyma. Further added CT features were laterality (left or right), absence or presence of collateral vascular supply defined as enlarged renal capsular veins that become macroscopically visible at CT or magnetic resonance imaging studies, infiltration, collecting system invasion, hydronephrosis, renal artery thrombosis, renal vein thrombosis, and intralesional hemorrhage [23]. Two additional CT features were perirenal fat stranding (absent or present) and Gerota’s fascia thickening (absent or present). Tumor size was acquired by measuring the maximum diameter of the tumor in the axial plane of the postcontrast images [23]. Collateral vascular supply was detected on the images acquired during the postcontrast phases. Well-defined margins were evaluated using a window with width (W) and level (L) values equal to 400 and 50, respectively, and considering a tumor circumference greater than 90% that appeared ‘pencil-thin’ sharp in the postcontrast images (including the interface with renal parenchyma, collecting system, and sinus and perinephric adipose tissue) [23]. When one or more well-delimited cystic spaces with fluid attenuation value, i.e., ≤20 Hounsfield Units (HU), was present in ≥50% of the tumor volume, the tumor was considered cystic [24]. The presence of the cystic component being <50% of the tumor volume or the absence of this component, defined the tumor as solid [23]. Tumor necrosis was evaluated by the presence of hypodense areas, lacking contrast enhancement, not sharply demarcated, and lacking apparent walls, radiologic features that allowed to differentiate it from the cystic component [23]. Tumor necrosis in solid tumors was evaluated during the nephrographic or excretory phases [23]. CT features of calcification were high-density spots or plaques. In doubtful cases of calcification, maximum HU values greater than 60 HU were considered as a cut-off [23]. Intralesional hemorrhage was detected by measuring HU within the tumor (i.e., HU blood +30 to +80). The differentiation of calcification and intralesional hemorrhage with similar HU was performed by two radiologists expert in oncologic imaging, on the basis of morphological characteristics (F.G., 8 years of experience; C.A.M., 12 years of experience) [23]. Cancer infiltration, assessed in postcontrast images, was characterized by the growth and development of tumor tissue into surrounding normal tissue [23]. This criterion was considered positive, after the assessment of the two radiologists, when the tumor tissue expanded from its site of origin to invade the local anatomical structures (e.g., psoas muscle, quadratus lumborum muscle or colon). Hydronephrosis, detected as dilatation of the urinary tracts, was assessed on postcontrast images [23]. Renal artery and vein thrombosis were characterized by observing the presence of thrombotic endoluminal filling defects of vessels on postcontrast images [23]. Collecting system invasion was detected by the presence of intraluminal filling defects of the collecting system, starting from the tumor, during the excretory phase [20].

### 2.4. Imaging Analyses

All patients underwent CT examination. CT images, obtained before and after intravenous administration of an iodine-based contrast medium, were analyzed for acquisition of CT feature data. Analysis of CT images was performed using Horos v.4.0.0 RC2 software. Each case was reviewed by two radiologists with experience in oncologic imaging (F.G., 8 years of experience; C.A.M., 12 years of experience), blinded to genomics data. Image size, window, and level setting were adjusted by the radiologists. Image analysis was performed in the axial and coronal planes, although all measurements were performed in the axial plane. Each reviewer used the predefined feature set independently, so that a score was assigned to each ccRCC.

### 2.5. Statistical Analyses

Descriptive statistics included frequencies and proportions for categorical variables. Medians and interquartile ranges (IQRs) were reported for continuously coded variables. Wilcoxon rank sum test, Pearson’s Chi-square test, and Fisher’s exact test examined the statistical significance of differences in medians and proportions among the patient cohort stratified according to GIMAP genes expression (yes versus no). All tests were two-sided with a level of significance set at *p* < 0.05. The R software environment for statistical computing and graphics (version 4.1.2, R foundation for Statistical Computing, Vienna, Austria) was used for all analyses.

A Wilcoxon rank sum test was used for continuously coded variables (age and primary tumor size), while both Pearson’s Chi-square and Fisher’s exact tests were used for categorical variables. Specifically, the former evaluated the statistical significance of differences in proportions for the following variables: sex, collateral vascular supply, tumor margins, calcifications, collecting system invasion, perinephric stranding, and Gerota’s fascia thickening; the latter examined the statistical significance of differences in proportions for the following variables: tumor grade, tumor stage, tumor composition, tumor necrosis, tumor growth pattern, signs of infiltration, hydronephrosis, thrombosis or infiltration of renal artery, thrombosis or infiltration of renal vein, and intralesional hemorrhage.

## 3. Results

**Figure 1:**
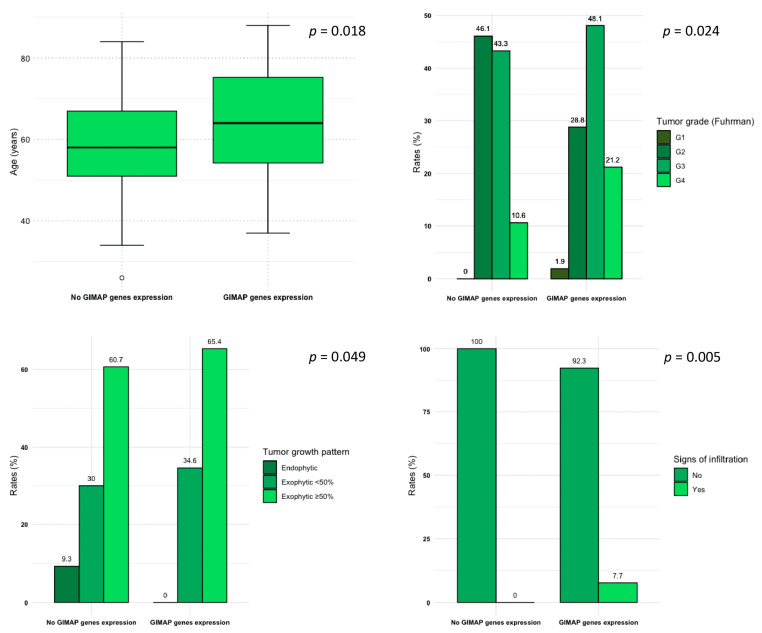
Box and whisker plots and bar plots depicting the distribution of age at initial diagnosis, Fuhrman tumor grade, tumor growth pattern, and signs of infiltration for the study population according to GIMAP genes expression.

In the overall cohort of 193 patients, 52 (26.9%) presented with GIMAP family genes expression (Table 2). Compared to patients who did not express GIMAP family genes, these subjects were older (median age: 64 [IQR 54–75] vs. 58 [IQR 51–70], *p* = 0.018), and more frequently presented with renal tumors of higher grades (21.2% vs. 10.6% for Fuhrman G4, and 48.1% vs. 43.3% for Fuhrman G3; *p* = 0.024), and a more advanced stage (19.2% vs. 9.3% for stage IV; *p* = 0.3) at final pathology. Radiologically, renal tumors in the GIMAP family genes expression patient group more frequently showed an exophytic growth pattern (100% vs. 90.7%; *p* = 0.049) or signs of infiltration (7.7% vs. 0%; *p* = 0.005) at the preoperative CT scan evaluation (see also Figure 1).

## 4. Discussion

**Figure 2:**
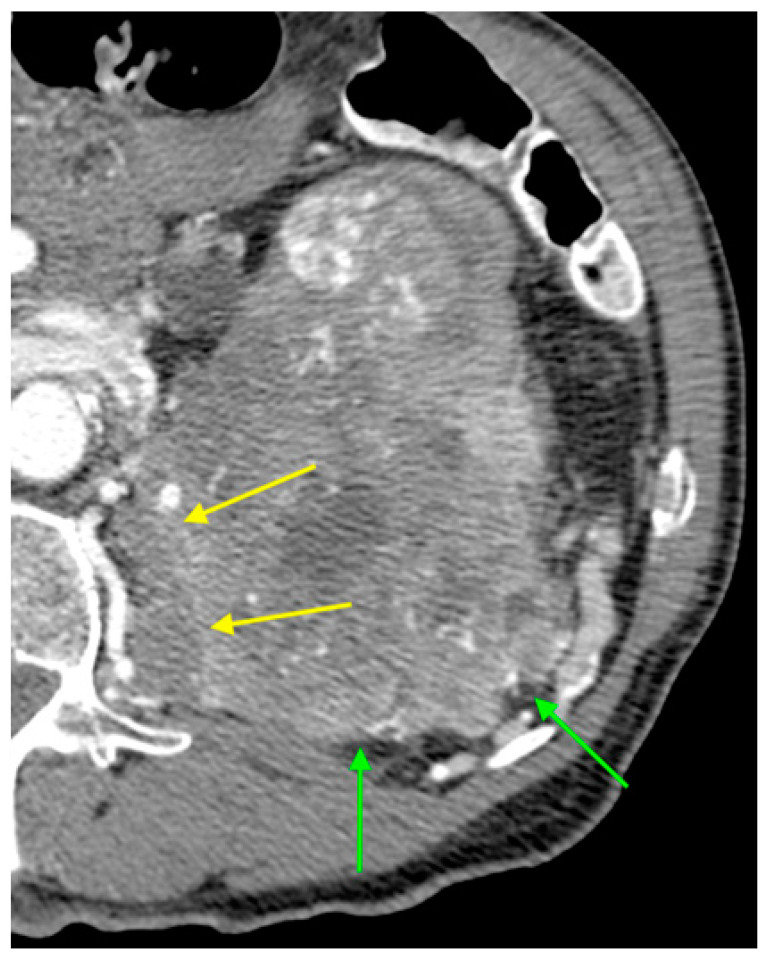
Axial CT image during arterial phase showing ccRCC with GIMAP genes expression with absence of endophytic growth pattern (green arrows) and signs of infiltration of the left psoas major muscle (yellow arrows).

In this study we evaluated CT features of GIMAP family genes in ccRCC patients. A significant association was found with absence of endophytic growth pattern (*p* = 0.049), presence of infiltration (*p* = 0.005), advanced age (*p* = 0.018) and high Fuhrman grade (*p* = 0.024). Absence of growth pattern and presence of infiltration are imaging phenotypic expressions of molecular manifestations of GIMAP family genes in ccRCC (Figure 2).

The abundant infiltration of immunocytes (in particular T cells) typical of ccRCC, characterizes it as an immunogenic tumor [24]. Macrophages, dendritic cells, CD4+ T cells, and CD8+ T cells are infiltrated in ccRCC [24]. GIMAPs are associated with immunity, as they regulate biological functions and states of multiple immunocytes. All of these proteins possess binding domains for GDP/GTP [17,18,20,21,25,26]. The amount of CD8+ T cells and the relative antitumor activity are significantly correlated with immunotherapeutic effect and clinical prognosis in tumors [27]. Cytokines secreted by ccRCC have been reported to influence the differentiation of dendritic cells, resulting in decrease or loss of antitumor activity of CD8+ T cells [28]. Mature dendritic cells are linked to the activation of CD8+ T cells and ccRCC’s favorable prognosis [29,30]. M1 macrophages, through the release of interleukin-12, interferon-γ, and tumor necrosis factor, determine the increase in the cytotoxic activity of CD8+ T cells [31]. GIMAP 1 and GIMAP 6 have a fundamental role in maintaining the quantity of CD8+ T cells [17,21]. A positive correlation of all GIMAP family members with CD8+ T cell infiltration in lung cancer has been demonstrated [32]. GIMAP7 also has a positive correlation with CD8+ T cell infiltration in pancreatic adenocarcinoma [33].

The assumption is that CT imaging features might be related to GIMAP expression. However, the correlation does not necessarily imply causation. Absence of endophytic growth pattern, presence of infiltration, and high Fuhrman grades are radiologic and pathologic features of aggressive disease. It is likely locally advanced ccRCC with GIMAP family genes expression in patients with advanced age are related to the activity of GIMAPs recruiting immunocytes into the TIME.

The presence of infiltration was also found in ccRCC with P4HA3 expression [23]. However, ccRCC with GIMAP genes expression presents a different radiogenomic pattern as the absence of endophytic growth pattern is a typical feature of this genes expression. Moreover, ccRCC with P4HA3 expression presents primary tumor size, ill-defined margins and more advanced tumor stage (American Joint Committee of Cancer), all features that are not present in ccRCC with GIMAP expression [23]. Radiogenomics of ccRCC has shown promising results in the correlation between gene expression and related radiological phenotypic pattern. The acquisition of these data through a non-invasive approach allows the collection of relevant prognostic data and possibly has important therapeutic implications aimed at the specific gene expression. Knowledge of genomics disease is critical for patient prognosis. For example, ccRCC with BRCA1-associated protein-1 (BAP1) mutation presents increased sensitivity to radiation therapy and greater sensitivity to the inhibitors of mammalian target of rapamycin complex 1 (mTORC1 inhibitors) [3,34]. Immunotherapy is an important therapeutic strategy for ccRCC, but only a small portion of these patients may benefit from this treatment [35,36,37]. Intratumoral proliferation of CD8+ T cells has been associated with improved clinical outcomes and response to immunotherapy; in fact, it has been demonstrated that the activity and quantity of CD8+ T cells increase after treatment with immunotherapy [14,38]. The efficacy of immune checkpoint blockade therapy could be due to the antitumor activity of antigen-specific CD8+ T cells [39,40]. Further studies will evaluate biological and molecular mechanisms of GIMAP family genes in ccRCC related to the phenotypic manifestations detected in this study and the correlation between GIMAP family genes expression in ccRCC and the proliferation of CD8+ T cells in TIME. Some *p*-values, like *p* = 0.049 for the endophytic growth pattern, are very close to the conventional significance threshold (*p* = 0.05). This might indicate only a weak association, and further studies on larger series might be needed to confirm the significance of these findings.

This study has some limitations: the retrospective nature of the study limiting the generalizability of the findings, lack of randomization, dependency on previously recorded data, lack of further data to analyze such as the survival outcome, the different number of patients included between two groups and the different age between the two groups.

## Figures and Tables

**Table 1:** GIMAPs localization and activity in human.

GIMAPs	Localization	Activity in Human
GIMAP 1 [17,18,19]	Golgi apparatus	Maintenance of T cells proliferation and mature B cells function
GIMAP 2 [19]	Lipid droplets	Not known
GIMAP 3 [19]	Endoplasmic reticulum	Not known
GIMAP 4 [19,20]	Cytosolic	May promote T cells apoptosis
GIMAP 5 [19]	Lysosomes and vesicles	Deficiency determines: T cells and natural killer cells defects and replicative senescence in T cells
GIMAP 6 [19,21]	Autophagosomes	Leads Jurkat T cells more susceptible to apoptosis inducers
GIMAP 7 [19]	Cytosolic	Not known
GIMAP 8 [19]	Not known	Not known

**Table 2:** Descriptive characteristics of the study population according to GIMAP genes expression.

Characteristic	Overall *n* = 193 ^a^	GIMAP Family Genes Expression		*p*-Value ^b,c,d^
		No *n* = 141 (73.1%) ^a^	Yes *n* = 52 (26.9%) ^a^	
**Clinical-pathological features**				
Age (years)	59 (51, 70)	58 (51, 67)	64 (54, 75)	**0.018 ^b^**
Sex (males)	131 (67.9%)	93 (66.0%)	38 (73.1%)	0.3 ^d^
Primary tumor size (mm)	52 (38, 78)	52 (37, 78)	55 (43, 71)	0.4 ^b^
Tumor grade (Fuhrman) G1 G2 G3 G4	1 (0.5%) 80 (41.5%) 86 (44.6%) 26 (13.4%)	0 (0%) 65 (46.1%) 61 (43.3%) 15 (10.6%)	1 (1.9%) 15 (28.8%) 25 (48.1%) 11 (21.2%)	**0.024 ^c^**
Tumor stage Stage I Stage II Stage III Stage IV	105 (54.7%) 18 (9.3%) 46 (24.0%) 23 (12.0%)	78 (55.7%) 15 (10.7%) 34 (24.3%) 13 (9.3%)	27 (51.9%) 3 (5.8%) 12 (23.1%) 10 (19.2%)	0.3 ^c^
**CT-based features**				
Collateral vascular supply	103 (54.8%)	78 (57.4%)	25 (48.1%)	0.3 ^d^
Tumor margins Ill-defined Well-defined	64 (33.3%) 128 (66.7%)	45 (32.1%) 95 (67.9%)	19 (36.5%) 33 (63.5%)	0.6 ^d^
Tumor composition Solid Cystic	176 (92.1%) 15 (7.9)	129 (92.1%) 11 (7.9%)	47 (92.2%) 4 (7.8%)	0.9 ^c^
Tumor necrosis 0% 1–33% 34–66% > 66%	12 (6.2%) 115 (59.9%) 45 (23.4%) 20 (10.5%)	9 (6.4%) 82 (58.6%) 34 (24.3%) 15 (10.7%)	3 (5.8%) 33 (63.5%) 11 (21.2%) 5 (9.5%)	0.9 ^c^
Tumor growth pattern Endophytic Exophytic < 50% Exophytic ≥ 50%	13 (6.8%) 60 (31.3%) 119 (61.9%)	13 (9.3%) 42 (30.0%) 85 (60.7%)	0 (0%) 18 (34.6%) 34 (65.4%)	**0.049 ^c^**
Calcifications	34 (17.8%)	22 (15.8%)	12 (23.1%)	0.2 ^d^
Signs of infiltration	4 (2.1%)	0 (0.0%)	4 (7.7%)	**0.005 ^c^**
Hydronephrosis	6 (3.1%)	3 (2.1%)	3 (5.8%)	0.3 ^c^
Thrombosis or infiltration of renal artery	4 (2.1%)	3 (2.2%)	1 (1.9%)	0.9 ^c^
Thrombosis or infiltration of renal vein	12 (6.3%)	9 (6.4%)	3 (5.8%)	0.9 ^c^
Collecting system invasion	58 (30.2%)	41 (29.3%)	17 (32.7%)	0.6 ^d^
Perinephric stranding	88 (70.4%)	64 (71.9%)	24 (66.7%)	0.6 ^d^
Gerota’s fascia thickening	59 (47.6%)	43 (48.9%)	16 (44.4%)	0.7 ^d^
Intralesional hemorrhage	4 (2.1%)	4 (2.9%)	2 (3.8%)	0.3 ^c^

^a^ Median (IQR); n (%) ^b^ Wilcoxon rank sum test; ^c^ Fisher’s exact test; ^d^ Pearson’s Chi-square test.

## Data Availability

Not applicable.
